# The Elusive Mitochondrial Genomes of Apicomplexa: Where Are We Now?

**DOI:** 10.3389/fmicb.2021.751775

**Published:** 2021-10-15

**Authors:** Luisa Berná, Natalia Rego, María E. Francia

**Affiliations:** ^1^Laboratory of Apicomplexan Biology, Institut Pasteur de Montevideo, Montevideo, Uruguay; ^2^Molecular Biology Unit, Institut Pasteur de Montevideo, Montevideo, Uruguay; ^3^Bioinformatics Unit, Institut Pasteur de Montevideo, Montevideo, Uruguay; ^4^Sección Biomatemática-Laboratorio de Genómica Evolutiva, Facultad de Ciencias, Universidad de la República, Montevideo, Uruguay; ^5^Departamento de Parasitología y Micología, Facultad de Medicina, Universidad de la República, Montevideo, Uruguay

**Keywords:** Apicomplexa, mitochondria, mitogenome, genome, *Toxoplasma*, malaria, PacBio and ONT

## Abstract

Mitochondria are vital organelles of eukaryotic cells, participating in key metabolic pathways such as cellular respiration, thermogenesis, maintenance of cellular redox potential, calcium homeostasis, cell signaling, and cell death. The phylum Apicomplexa is entirely composed of obligate intracellular parasites, causing a plethora of severe diseases in humans, wild and domestic animals. These pathogens include the causative agents of malaria, cryptosporidiosis, neosporosis, East Coast fever and toxoplasmosis, among others. The mitochondria in Apicomplexa has been put forward as a promising source of undiscovered drug targets, and it has been validated as the target of atovaquone, a drug currently used in the clinic to counter malaria. Apicomplexans present a single tubular mitochondria that varies widely both in structure and in genomic content across the phylum. The organelle is characterized by massive gene migrations to the nucleus, sequence rearrangements and drastic functional reductions in some species. Recent third generation sequencing studies have reignited an interest for elucidating the extensive diversity displayed by the mitochondrial genomes of apicomplexans and their intriguing genomic features. The underlying mechanisms of gene transcription and translation are also ill-understood. In this review, we present the state of the art on mitochondrial genome structure, composition and organization in the apicomplexan phylum revisiting topological and biochemical information gathered through classical techniques. We contextualize this in light of the genomic insight gained by second and, more recently, third generation sequencing technologies. We discuss the mitochondrial genomic and mechanistic features found in evolutionarily related alveolates, and discuss the common and distinct origins of the apicomplexan mitochondria peculiarities.

## Introduction

Mitochondria are distinctive double-membrane organelles of central importance to the biology of eukaryotic cells. Mitochondria are ubiquitous through the eukaryotic domain. It is accepted that the last common ancestor of extant eukaryotes was a mitochondrion-containing organism generated by an event of endosymbiosis following the integration of an alpha-proteobacterium into a cell related to Asgard archaea (Roger et al., 2017). Classically, mitochondria are regarded as the prime energy conversion hubs in aerobic organisms, as a product of the electrochemical potential generated along the respiratory chain. However, it is well established that mitochondrial functions extend well beyond respiration and energy conversion, impacting other biological processes central to life. These include the participation of mitochondria in apoptosis, amino acid metabolism, calcium homeostasis, pyruvate decarboxylation, folate, phospholipids, heme, and iron-sulfur clusters biosynthesis, among others (Maguire and Richards, 2014; Santos et al., 2018). Many of these functions are performed either by proteins coded for in the nucleus and imported into the mitochondrion, or by proteins coded for in the mitochondrial genome. The former arose mainly by past lateral gene transfer from the mitochondrion to the nucleus and repurposing and/or redirecting pre-existing nuclear genes. Anaerobic and facultative aerobes harbor functionally reduced and structurally simplified mitochondrion-related organelles, generally referred to as MROs. These include the hydrogenosomes and the mitosomes. In some cases mitochondria are no longer observable as a product of a secondary loss. In these cases, the lack of mitochondria can be tolerated by the novel acquisition of a functionally compensating cytosolic metabolic pathway. This is possible, in some instances, by lateral gene transfer from bacteria. Mitochondrial reduction accompanied by specific alterations in metabolic capabilities is often connected to adaptation to specific ecological niches (Seeber et al., 2008; Karnkowska et al., 2016).

Unlike other membrane bound organelles, with the exception of plastids, mitochondria bear their own extranuclear genome. The mitochondrial genome, from hereon referred to as “mitogenomes” can encode for tens of proteins, including those required for its maintenance (i.e., its replication). However, the coding information varies widely in nature. It can encompass part of the translation apparatus, including mitochondrial-specific tRNAs, large and small ribosomal RNAs (*rns* and *rnl)*; membrane associated proteins which catalyze oxidative phosphorylation: cytochrome b (*cob*), subunits of cytochrome c oxidase (*cox*), ATP synthase subunits 6, 8, and 9, subunits of the NADH dehydrogenase complex; and a few additional ORFs of unknown function. However, examples of mitogenomes displaying coding reduction are readily found in nature. Mitogenome sizes can vary widely in nature, ranging from 6 kb in protists to 2,400 kb in angiosperms.

Animal mitochondrial genomes exhibit an exceptional circular-supercoiled DNA topology (Lecrenier and Foury, 2000). This topology can also be found in kinetoplastids (Simpson, 1986). However, classical pulsed-field electrophoresis experiments from the 90’s showed that most commonly, mitogenomes of plants and fungi exist in linear topologies (Bendich, 1993, 1996). Linear configurations are found as concatemers of monomeric subunits (Burger et al., 2003), arranged in head to head or head to tail configuration (Oldenburg and Bendich, 2001). Linear combinations of distinct subunits and *bona fide* linear monomeric mitogenomes have been documented. Illustrative examples of the former include the mitogenomes of the ciliate *Tetrahymena pyriformis* (Burger et al., 2000) and the algae *Chlamydomonas reinhardtii* (Gray and Boer, 1988).

The phylum Apicomplexa comprises over six thousand protozoan species of unicellular obligate intracellular parasites. Infamous pathogens causing an immense burden of mortality and morbidity, in both humans and animals, belong to this phylum. These include the causative agents of malaria, cryptosporidiosis, East Coast fever, tropical theileriosis, babesiosis and toxoplasmosis, among others. Though the pathobiology of apicomplexan diseases are notably distinct, parasites belonging to the phylum share a number of conserved structural features which underlie their pathogenesis. Namely, apicomplexans owe their name to a complex of apical secretory organelles equipped with effectors, adhesins and virulence factors that are hierarchically and sequentially deployed to allow recognition, invasion and subversion of their preferred host cell type (Dubremetz, 1973). In most species, each individual apicomplexan cell is equipped with a non-photosynthetic plastid remnant and a single, ramified mitochondrion, morphologically characterized by a dense matrix and inner mitochondrial membrane folds (cristae) with a circular profile (de Souza et al., 2009). Beyond these shared features, apicomplexans constitute a phylum of diverse organisms, showcasing an array of fascinating biological adaptations to the parasitism of specific niches. In this context, the presence, morphology and complexity of endosymbiotic organelles -the mitochondrion and the apicoplast- vary widely within lineages and between species.

The detailed study of the Api-mitochondrion and its complexity has been driven by the demonstration that Complex III of the mitochondrial electron transport chain (mETC) is a major drug target of the antimalarial atovaquone (Korsinczky et al., 2000; Mather et al., 2007; Vaidya et al., 2008; Biagini et al., 2012; Goodman et al., 2017; Pan et al., 2017). More recently, genome-wide genetic screens have deepened the interest. Genes coding for mitochondrial functions rank amongst the most important in conferring fitness, demonstrating the organelle’s vital role in the survival of the causative agent of human malaria, *Plasmodium falciparum*, and of toxoplasmosis, *Toxoplasma gondii* (Sidik et al., 2016; Bushell et al., 2017). Complexome analyses have recently shown that the major mitochondrial complexes in *P. falciparum* and *T. gondii* are formed by a combination of conserved and divergent protein components further putting the organelle forward as a promising source of much sought-after novel drug targets (Huet et al., 2018; Salunke et al., 2018; Seidi et al., 2018; Hayward and van Dooren, 2019; Evers et al., 2021; Maclean et al., 2021).

Recent sequencing analyses using third generation sequencing, such as Pacific Bioscience (PacBio) and Oxford Nanopore Technologies (ONT) have revealed a highly variable, and unusual mitochondrial genome organization in a number of medically relevant apicomplexans. However, the contribution of the mitochondrial genome to the biology of Apicomplexa remains unclear. Apicomplexan parasites constitute a heterogenous group of fascinating model organisms ideally suited to explore and understand the diversity and functional significance of distinct configurations of the mitochondrial genome. Apicomplexan mitochondria also represent extraordinary examples of selective retention, loss and gain, depicting unique features of evolution within the alveolates. This review summarizes the structure of apicomplexan mitochondrial genomes as currently understood. We highlight the unique and conserved apicomplexan mitogenome features, in the light of classical ultrastructural and biochemical insight, and emerging understanding brought by third generation sequencing technologies and single cell genomics.

## Phylogeny of the Phylum Apicomplexa

The currently accepted phylogenetic classification of Apicomplexa was originally proposed by Levin in 1970. Based on electron microscopy studies from the 50’s, this phylogeny contributed to reclassifying organisms originally grouped together under the term “Sporozoa.” The apicomplexans were grouped based on the presence of a visually detectable apical complex. Under Levin’s classification, groups of organisms such as Microspora, Myxozoa, and Ascetospora, were separated from Apicomplexa based on their distinct morphologies (Levine, 1988). The advent of molecular techniques in the 90’s allowed insight into genomes and further refinement of this classification. Apicomplexans belong to the superphylum Alveolata, which they share with Chrompodellids, Perkinsozoa, Dinoflagellata, and Cilliophora.

Emerging data based on single-cell genomics of apicomplexan groups suggests that the currently accepted phylum, encompassing over 6,000 species, could actually be polyphyletic (Janouškovec et al., 2019; Mathur et al., 2021). In light of these results, it has been put forward that morphological similarities could have evolved convergently at least three times. Nonetheless, taxa important from a medical and veterinary perspective are all classified and grouped within Apicomplexa *sensu stricto* (Janouškovec et al., 2019), where species are further classified as coccidiomorphs, gregarines and cryptosporidians. Coccidiomorphs include species classified as agamococcidians, Coccidia and Hematozoa; arguably the most intensely studied and best understood species within the phylum belong to the latter two groups. Coccidians can be further classified as eucoccidians and protococcidians. Eucoccidians can be further classified as either “cyst forming” or monoxenic. The former includes *T. gondii*, *Neospora caninum, Hammondia hammondi, and Sarcocystis*, while the latter encompasses the family Eimeriidae. Eimeriidae is composed of multiple important genera, including *Eimeria*, *Isospora*, and *Cyclospora*. Species within these genera cause gastrointestinal disease, and characteristically complete their life cycle within a single host’s intestinal tract.

**FIGURE 1:**
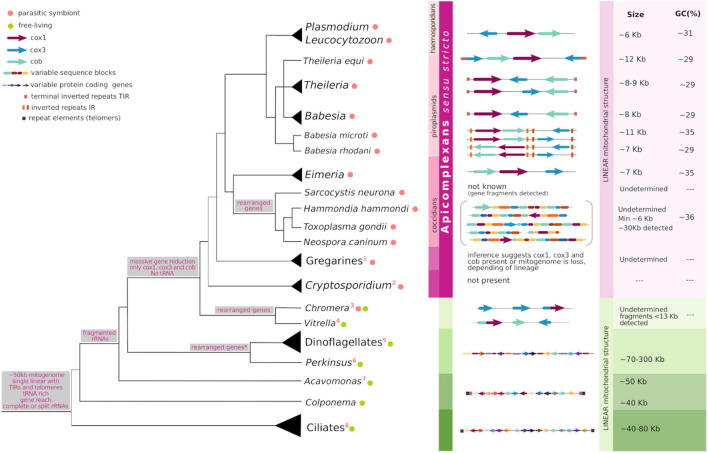
Commonalities and peculiarities of apicomplexan mitogenomes in light of their evolution. The tree shows evolutionary relationships among taxa in Alveolata, the gray branches at the base of Alveolata and Apicomplexa depict less confidence for the topology shown. Gray boxes associated with some tree nodes describe the characters present or lost at the hypothetical common ancestors. To the right of taxon names, colored circles indicate the organism lifestyle following the legend and numbers enumerate peculiar features of each lineage (see below). At the middle, the horizontal figures represent the size, topology and gene content of known mitogenomes. The different elements follow the shape and color depicted in the legend. To the right, when the data is available, approximated mitogenome size and GC content (%) are indicated. This figure results from an adaptation of Figure 7 in Namasivayam et al. (2021), Figure 5 in Hikosaka et al. (2010), Figure 5 in Schreeg et al. (2016), and Figure 1 in Janouškovec et al. (2019). 1: mitogenome is lost in some gregarines; 2: mitogenome loss; 3: *cox1–cox3* fusion, *cob* loss; 4: *cob-cox1* fusion; 5: fragmented *cox3* and *cob-cox3* fusion, extensive RNA editing; 6: *cox3* loss and translational frameshifting in *cox1*; 7: *cox2* encoded for in the mitogenome; 8: bipartite rRNAs, few tRNAs, *cox3* loss, bipartite *nad1*.

Hematozoa can be further classified into Haemosporida; the malaria causing species of *Plasmodium* belong to this group, and Piroplasmida which includes *Theileria* and *Babesia* spp. Finally, the cyrptosporidians include all *Cryptosporidium* species; *C. hominis, C. parvum, C. muris*, among others. Recently, transcriptomic data allowed the assessment of the divergence pattern involving coccidiomorphs, gregarines and cryptosporidians and results support the placement of *Cryptosporidium* as the earliest diverging lineage of apicomplexans (Mathur et al., 2021; Salomaki et al., 2021). However, the history of early apicomplexan radiation remains highly contentious (Janouškovec et al., 2019; Mathur et al., 2019, 2021; Salomaki et al., 2021), which impacts our interpretation of evolutionary events within the phylum. Figure 1 summarizes our current understanding of the phylogeny of alveolates.

## The Mitochondria of Apicomplexans

Electron microscopy from the 1960s clearly identified double membrane-bound mitochondrial organelles in many apicomplexans including *Plasmodium, Toxoplasma*, and *Eimeria* (Aikawa et al., 1966; Hepler et al., 1966; Rudzinska, 1969; Divo et al., 1985; Slomianny and Prensier, 1986; Ferguson and Dubremetz, 2014). However, it was noted that the cristae morphology differed from that found in mammals, and that there is a single mitochondrion per cell. Both in *Toxoplasma* and in *Plasmodium*, cristae are bulbous in shape and not lamellar (as are in mammals) (Aikawa et al., 1969; Ferguson and Dubremetz, 2014; Muhleip et al., 2021). The proper formation of hexamers of the F1F0 ATP synthase residing in the cristae membrane of the *T. gondii* mitochondria was shown to be critical for the maintenance of the native cristae morphology (Muhleip et al., 2021). However, tubular cristae morphology is conserved in gregarines whose expression of the ATP synthase genes is not detectable. This suggests that alternative cristae formation mechanisms could operate in Apicomplexa (Mathur et al., 2021).

The localization and morphology of the organelle vary as the cell cycle progresses (Melo et al., 2000; Dooren et al., 2006; Ovciarikova et al., 2017). The non-dividing parasite mitochondrion is generally elongated or branched (Melo et al., 2000; Dooren et al., 2006; Ovciarikova et al., 2017). However, as cell division onsets, mitochondria undergo marked morphological changes to accommodate the specific requirements of distinct cell division mechanisms used by each species. For instance, the *Plasmodium* mitochondrion replicates its DNA coincidentally with nuclear DNA replication (Preiser et al., 1996). In the process, it becomes highly branched during schizogony; a cell division mechanism which generates tens of emerging daughter cells simultaneously. The mitochondrion divides into multiple organelles late during schizogony, prior to cytokinesis, at the time in which other organelles such as the nucleus are packed (Dooren et al., 2006). Mitochondrial duplication starts at the early stages of daughter formation in *Toxoplasma*’s endodyogeny. The mitochondrion branches at multiple locations, entering the developing daughter cells. In the cell division scheme used by *T. gondii* two daughter cells are formed at a time; this mechanism is known as endodyogeny. Here, again packing of the mitochondria into the new parasites happens at the very end of the division cycle (Nishi et al., 2008; Ovciarikova et al., 2017; Melatti et al., 2019).

In *T. gondii*, mitochondrial segregation has been further elucidated. Two proteins, Fis1, a homolog of Fis1p protein of yeast, and LMF1, an outer mitochondrial membrane protein, were shown to interact with each other. Fis1 homologs play a role in recruiting fission-mediating dynamins in other systems, such as bacteria. Disruption of LMF1 in *T. gondii* severely impacts mitochondrial morphology, and impairs proper organellar segregation (Jacobs et al., 2020). In all cases, however, little is known about the mechanism of mitochondrial genome duplication and maintenance and its coordination with cell cycle progression.

A remarkable exception regarding the biology of mitochondria among Apicomplexa is *Cryptosporidium*. While all others in the phylum retain the mitochondria, cryptosporidians possess a double membrane remnant organelle which has lost its metabolic capability, generally referred to as the mitosome (Putignani et al., 2004). In contrast to other mitochondrial reductions found in nature, as for example hydrogenosomes, the mitosome represents an additional level of reduction as it does not retain any genetic material (Tachezy, 2008; Hjort et al., 2010). The mitosome evolutionary history can only be traced thanks to the localization of resident mitochondrial proteins, such as the mitochondrial chaperonin 60 (Cpn60) (Riordan et al., 2003) and mitochondrial heat shock protein 70 (HSP70) (Šlapeta and Keithly, 2004) to the organelle. The loss of the mitochondrion is accompanied by the loss of the plastid remnant and a reduction of the nuclear genome. It is thought that the abundance of host-derived nutrients combined with anaerobic/hypoxic growth conditions support the massive reduction in the mitochondrial metabolic capability in cryptosporidians (Gagat et al., 2017). Two additional independent mitochondrial functional reductions and electron chain losses within Apicomplexa have been recently shown to have occurred amongst gregarines (Mathur et al., 2021; Salomaki et al., 2021). While these mitochondrial reductions might be related to the hypoxic conditions found within the digestive tract of their respective hosts, further studies are needed to proper characterize these organelles.

## Api-Mitochondrial Genome Sizes and Topological Organization

A common theme to Myzozoa, including chromerids, dinoflagellates, perkinsids, and Apicomplexa *sensu stricto*, is the extreme reductive evolution of their mitogenomes. A hallmark of these genomes is that they are stripped down to the bare minimum, retaining only the coding information required to continue existing as independent entities. Most myzozoans retain at most three protein coding genes; apocytochrome b (*cob*) and two subunits of cytochrome c oxidase (*cox1* and *cox3*). Mitogenomes also include fragmented genes for ribosomal RNAs of the Large and Small subunits (LSU and SSU fragments, respectively), and other fragments of unknown function. No mitochondrial tRNA genes are present and, as it is understood, their loss is compensated by functional replacement with cytosol-imported tRNAs (Esseiva et al., 2004; Pino et al., 2010). In stark contrast, sister lineages to myzozoans, such as Acavonomidia and Colponemidia, include over 45 protein coding genes in their mitochondrial genomes. Of these, nine were transferred to the nucleus in Apicomplexa (including the cytochrome c oxidase 2 gene), while 30 were presumably lost. One hypothesis for the selective retention of the three protein-coding genes in the myzozoan mitogenomes argues that given the key roles played by these proteins in electron transport and energy coupling, their expression must be quickly and directly regulated by the redox state of the mitochondrion (Allen, 1993; Adams and Palmer, 2003; Martin, 2003). Additionally, GC content and protein hydrophobicity have been proposed as factors favoring mitochondrial retention (Johnston and Williams, 2016). In fact, the hydrophobicity-related hypothesis was the first to be proposed based on the rationale that hydrophobic segments present in mitochondrial proteins could potentially target them for transport to the endoplasmic reticulum, thus preventing import across the mitochondrial membrane (von Heijne, 1986). Experimental evidence and additional observations have contributed to further support this mitochondrial-targeting hypothesis (Björkholm et al., 2015; Muhleip et al., 2021). Concordantly, the three retained genes, which are almost universal among currently examined mitochondrial genomes, exhibit highly hydrophobic profiles. Importantly, three other respiratory genes, *nad1*, *nad4*, and *nad5*, are present in all mitogenomes except for a number of alveolates, including all apicomplexans, and some yeasts. In fact, what is missing is the entire NADH dehydrogenase complex (composed of over 20 subunits) known as Complex I of the electron transfer chain (Adams and Palmer, 2003; Vaidya and Mather, 2009; Flegontov et al., 2015). In such a context, organisms rely on alternative NADH dehydrogenases (NDH2) to transfer electrons to ubiquinone (Biagini et al., 2006; Flegontov et al., 2015; Hayward and van Dooren, 2019). In absence of Complex I, Complex III is the first proton pumping complex in the apicomplexan mETC.

Dinoflagellates have increased their mitogenome size by accumulating a surprisingly high (99%) percentage of non-coding and repetitive sequence elements, reaching mitogenome sizes of up to 300 kb. It has been proposed that these sequence additions might be functional and positively selected in free-living organisms subject to more stringent metabolic conditions than those which have remained exclusively parasitic and under a scenery of relaxed selection (Gagat et al., 2017). Comparatively, mitogenomes in Apicomplexa are generally small, with the largest one characterized to date being of less than 30 kb, and the majority being smaller than 12 kb (Figure 1). The mitochondrial genomes of haemosporidians (*Plasmodium* and *Leucocytozoon*), piroplasmids (*Babesia, Theileria* and archaeopiroplasmids) together with Eimeriidae coccidians (*Eimeria, C. cayetanensis*, and *Isospora)*, are amongst the smallest characterized in nature, ranging from 5 to 12 kb in size (Hikosaka et al., 2010; Cinar et al., 2015; Hafeez and Barta, 2019). Nonetheless, no correlation *per se* has been established between the mitochondrial genome size and its gene content. This is clearly illustrated by the fact that ciliates, whose mitogenomes encode for tens of proteins usually range from 50 to 70 kb in size while dinoflagellates bear mitochondrial genomes of about 300 kb, but conserve only three protein coding genes (Burger et al., 2003; Gagat et al., 2017; Park et al., 2019). Variations in genome sizes are mostly caused by repetitive elements, and differences in the length and organization of intergenic regions.

One of the best characterized mitochondrial genomes in the phylum Apicomplexa belongs to *Plasmodium.* As for many other aspects of their cell biology, our disproportionate insight into the -omics of *Plasmodium* species is likely a consequence of their tremendous impact to human and animal health. Malaria remains one of the most deadly infections affecting millions of people worldwide, yearly. This interest is exacerbated for the *Plasmodium* mitogenome, as the organelle has been identified as the target of the antimalarial drug atovaquone, and of other drugs showing antimalarial effects *in vitro* (Vaidya et al., 1993). The first molecular insights into the mitogenome in *Plasmodium* inadvertently emerged in the late 80’s when “the 6 kb element” was first identified in the murine and human infecting species *P. yoelii* and *P. falciparum* (Vaidya and Arasu, 1987; Vaidya et al., 1989), respectively.

The “6 kb element” initially attracted interest due to its repetitive nature (Suplick et al., 1988). However, the presence of an additional extrachromosomal DNA element -which we know now corresponds to the remnant plastid genome- confounded its conclusive identification (Feagin, 1994). Definitive proof came from sequencing experiments which demonstrated that this element coded for three classical mitochondrial proteins (Suplick et al., 1990; Feagin, 1992). Early sequencing experiments of the 6 kb element in different *Plasmodium* species showed that both the sequence and the physical arrangement of the mitogenome are highly conserved. The fast paced advances in sequencing technologies exponentially increased the number of sequences available: by 2011, complete or near-complete mitochondrial genome sequences were already available for 25 species of *Plasmodium* (Hikosaka et al., 2011b), consolidating the notion that the organizational principle of the 6 kb element is universal to the mitogenomes of *Plasmodium* species. The 6 kb element was estimated at the time to exist in about 20 copies per cell in *P. falciparum*. This coincides with the copy number reported for *P. gallinaceum* (15–20/cell) (Joseph et al., 1989), but is markedly lower than the number of copies reported for *P. yoelii* (150/cell) (Vaidya and Arasu, 1987). The biological significance of these differences remains unclear.

The topology of the mitogenome in *Plasmodium* was initially proposed to be a combination of circular and linear molecules. Still, this combination is actively reported in the literature. However, sequencing and biochemical assays corroborated the identity, topology and size of *Plasmodium* mitogenome. Hybridization experiments elegantly demonstrated that the 6 kb element did not exist as a linear monomer, but rather exists within tandem arrays of varying sizes. These tandems range from 6 to 30 kb, with a marked preponderance of dimers of 12 kb (Joseph et al., 1989). Preiser and colleagues showed in 1996, through fine analyses of the mitogenome configuration of *P. falciparum*, using 2D gels, that circular topologies observed by electron microscopy of isolated mitochondrial DNA constitute a minor fraction of the molecules. These data reinforced the notion that the vast majority of DNA molecules of the mitogenome exist in linear configuration (Preiser et al., 1996).

Mitochondrial genomes have been looked into in a number of other haemosporidians belonging to three genera distinct from *Plasmodium*: *Leucocytozoon*, *Haemoproteus* and *Hepatocystis*. The mitogenomes of *L. caulleryi*, *L. fringillinarum*, and *L. majoris* share both size and organization with *Plasmodium*. Similarly, analyses of mitogenomes of seven *Haemoproteus* spp., two *Hepatocystis* spp., and 24 *Parahemoproteus* spp. support the notion that the mitogenome conservation extends throughout the order Haemosporida (Seeber et al., 2014; Pacheco et al., 2018). However, contradicting reports exist when it comes to the mitogenome sequences of bat parasites of the *Nycteria* genus; also haemosporidians. Karadjian et al. (2016) showed that in these species mitogenomes exhibit two distinct organizations; while *N. gabonensis* and *N. heischi*, display the typical haemosporidian sequence and organization found in *Plasmodium*, *N. medusiformis*, and *Nycteria* sp. (isolated as mixed infection), differ in that the *cob*, *cox1*, and *cox3* genes appear in a different order and transcriptional direction. In addition, the pattern of fragmentations of the large- and small-subunit rRNA genes is distinct. Though no evidence of such genome rearrangements were found for other species in over 100 mitogenome analyses, these results suggest that subtle differences may exist. Though the actual diversity in organization remains slightly controversial in Haemosporida, data disproportionally supports a highly conserved topology, and in all cases it is clear that all mitogenomes abide by the linear “6 kb element” monomeric subunit rule.

The mitogenomes of several Babesia and Theileria are composed of linear molecules characterized by the presence of large terminal inverted repeats on both ends (Hikosaka et al., 2010). However, differences in topology and organization exist between species and within lineages. The first sequenced mitochondrial genomes among the piroplasmids showed conservation in the structure and size within the order.

Within the genus *Babesia* the mitogenome structure is highly conserved. The mitogenomes of *B. bovis, B. bigemina, B. caballi, B. orientalis*, and *B. gibsoni* are made up of monomeric linear molecules of 6.6 kb characterized by the presence of flanking terminal inverted repeats (TIRs) of 440 bp (Hikosaka et al., 2010; He et al., 2014). Recently, mitogenomes for *B. canis*, *B. rossi*, *B. vogeli*, *B. gibsoni*, *Babesia sp. Coco*, and *Cytauxzoon felis* were characterized, revealing that they share the organization reported in *Babesia sensu stricto* and *Theileria* species (Schreeg et al., 2016; Guo et al., 2019). Nonetheless, differences are also identifiable; the presence of the repeated terminal TIRs and a *cox1* segment for *B. vogeli* and *Babesia sp. Coco* could not be confirmed. Likewise, the mitochondrial genome of *B. conradae* presents a novel arrangement of its genes, including the loss of *cox3* and a duplicated inversion that includes the 3′ end of *cox1* and ribosomal gene fragments (Schreeg et al., 2016).

Whole genome sequencing of *B. microti* R1 and Gray strains supported a previous notion based on 18S rRNA sequence, that it defines a basal branching new lineage within Piroplasmida, different from *Babesia* and *Theileria* (Cornillot et al., 2012). Genome mapping of these *B. microti* strains revealed that the mitochondrial genome is made up of four distinct linear molecule configurations in this species. Similar findings were reported for *B. microti* Munich strain, and for *B*. *rodhaini* (Hikosaka et al., 2012). These archeapiroplasmid mitogenomes present two pairs of novel inverted repeats -named IRa and IRb- and a flip-flop inversion in between. Combination of distinct pairs generates four distinct mitogenome structures present at an equi-molar ratio (Hikosaka et al., 2012). All possible configurations are similarly organized in linear molecules, however, mitogenome sizes differ amongst archeapiroplasmids; *B. microti* mitogenome is 11.1 kb while that of *B. rodhaini* is 6.9 kb. For both species, it was shown that multiple forms of the mitogenome coexist within a mitochondrion, displaying a total of about 20 copies per cell (Hikosaka et al., 2012).

While a number of *Theileria* species, such as *T. parva* and *T. annulata*, display mitogenomes very similar to those found in *Babesia* (Kairo et al., 1994; Hikosaka et al., 2010; Cornillot et al., 2013), sequence variations are more frequently observed. For instance, *T. orientalis* has a 3 kb central inversion including *cox3* and LSU fragments flanked by distinctive insertions (Hikosaka et al., 2010). *T. ducani* presents inverted repeats of only 48 bp at both ends (Virji et al., 2019). *T. equi* presents the most divergent mitochondrial genome of the theilerids displaying evidence of gene duplication and rearrangements, larger and more divergent TIR sequences which include *cox3* and rRNA gene fragments, resulting in a longer mitogenome molecule of 9 kb in size (Hikosaka et al., 2010; Kappmeyer et al., 2012). Similarly to *B. microti*, the phylogenetic position of *T. equi* is not clear and, while these features are consistent with an early branching of these species within Piroplasmida, a better understanding of the evolutionary patterns within this group requires further and better understanding of the group’s systematics.

Regarding Eimeriidae, varying size mitochondrial DNA fragments of 4–20 kb were observed by Southern blots when probing for the mitochondrial *cox3* gene of *E. tenella* (Hikosaka et al., 2011a). Consistently, the *E. tenella* mitogenome was shown to be arranged in tandemly repeated linear 6.2 kb elements (Hikosaka et al., 2011a). This structure and the mitogenome organization was later shown to be highly conserved amongst species of *Eimeria*. In fact, the sequencing of *E. mitis* (Liu et al., 2012), *E. magna* (Tian et al., 2015) and seven other species responsible for coccidiosis in turkey (Ogedengbe et al., 2014; Hafeez and Barta, 2019), revealed that mitogenomes are more stringently conserved among these species (displaying 0–0.5% variation) than their nuclear genomes are (1.6–3.2%) (Ogedengbe et al., 2014), suggesting they are under stricter constrain to change.

*Cyclospora cayetanensis* is another human pathogen of emerging importance within Eimeriidae. Its mitogenome has been shown to be formed by concatemers of a monomeric 6.3 kb subunit (Cinar et al., 2015; Ogedengbe et al., 2015; Tang et al., 2015). Parasites of the genus *Isospora*, composed of mostly avian and reptile parasites, display an akin mitogenome organization to that of *C. cayetanensis* and others within Eimeriidae. The mitogenomes of *I. serinuse, I. manorinae, I. amphiboluri* and an unnamed *Isospora* sp. have been shown to be of about 6 kb in size. The latter two were reported to be circular-mapping but their physical structure was not determined; circular mapping molecules plausibly suggest a topology of linear concatemers. Gene arrangement and directions are identical to that of other mitogenomes from *Eimeria* spp. and others in the family (Yang et al., 2017; Hafeez and Barta, 2019).

In contrast to the abundance of detailed information available for *Plasmodium* and its close relatives, and the relatively good understanding of the mitogenomes of monoxenic coccidans, cyst forming coccidians are still in the works. Early attempts to assemble these mitogenomes were presumably hampered due to the presence of fragmented copies of mitochondrial genes, especially *cob* and *cox1*, in the nuclear genome. This, has been argued, precluded the definitive identification of *bona fide* mitochondrial sequences. Using PacBio and ONT sequencing technologies, we and others recently attempted to assemble the mitochondrial genomes of both *T. gondii* and *N. caninum*. Contigs belonging to the mitochondrion could be readily identified by their markedly lower GC content (approximately 36%). Unexpectedly, however, neither we nor others embarking in similar efforts could assemble the tens of different mitochondrial contigs and reads into a single mitogenome. Contigs corresponding to the mitogenome vary widely in size. We found the mitogenome of *N. caninum* to be distributed in 20 distinct contigs, ranging from 1.4 to 86 kb with a mean of 12 kb in size (Berná et al., 2021). Twenty nine contigs ranging from 1.1 to 39 kb in size, with a median of 8.0 kb, were identified as the mitochondrial genome of *T. gondii* (Berná et al., 2021).

Consistently, Namasivayam et al. (2021) reported ONT reads of variable size ranging from 320 to 23,619 bp for *T. gondii*. This study carefully analyzed individual sequencing reads, revealing that though the composition of each read is not equal, nor is it made up of linear arrangements of a unique monomeric subunit, discrete common short sequence elements can be identified. Twenty one of these elements, referred to as “sequence blocks” named after the alphabet (A to V), appear in a plethora of arrangements in molecules of varying total size. These variable combinations give rise to an extraordinarily complex genome sequence. However complex, these authors discovered that the sequence blocks do not assemble randomly (Namasivayam et al., 2021). Rather, blocks appear in a finite number of combinations. As an illustrative example, *cox1* fragments are only present in sequence blocks V, S, C, and Q. The correct order combination of these results in a nearly full length *cox1*. Astoundingly, the reads representing the *T. gondii* mitogenome identified by Namasivayam et al. (2021), and the contigs assembled by us, differ significantly (though sequence blocks are clearly identifiable in both), suggesting the saturating sampling of possible combinations has not been reached even by the sum of our sequencing efforts. The mitogenome of *H. hammondi* seems to abide by the same principles of complexity both in size and organization as those of *T. gondii* and *N. caninum*, suggesting this composition complexity is a common feature of cyst-forming coccidia mitogenomes (Namasivayam et al., 2021).

A partially assembled genome of *S. neurona* has been produced (Blazejewski et al., 2015), showing an unusually large nuclear genome size, twice as large as other coccidian genomes, and with a repeat content approximately five times that of *T. gondii*. A definitive mitogenome has not been identified. However, analyses of publicly available *Sarcocystis* sequence reads (Blazejewski et al., 2015) by us (unpublished) and Namasivayam et al. (2021), clearly identify variable size contigs, ranging from 500 to 1,300 bp, whereby mitochondrial gene fragments can be identified. This suggests that the complexity of the mitogenome in these species might be akin to that of other cyst forming coccidians.

Though recent studies using third generation sequencing have allowed unequivocal identification of *bona fide* mitogenome sequences, overcoming the limitations of earlier technologies, the definitive size and topology of the elusive mitogenomes of cyst forming coccidians continues to be an enigma. Nonetheless, it should be noted that generally speaking, mitogenome sequencing can be influenced by low sequencing depths caused by low abundance of starting material (derived from difficult to culture parasites bearing a single copy of the organelle). In addition, protocols to obtain pure mitochondrial fractions from many species are lacking, and have only recently been optimized and used for organelle proteome profiling in a few species (Evers et al., 2021; Maclean et al., 2021). These fractions, however, are usually contaminated with plastid DNA. These limitations, combined with the use of analysis strategies based on mapping short-read sequences to highly repetitive DNAs, pose specific technological limitations which may influence the outcomes, either generating *de novo* artifactual assemblies, or missing subtle differences by inadvertently forcing synteny.

## Api-Mitochondrial Genome Transcription and Translation

Mitochondrial components are generally encoded by the nucleus while the organelle genome encodes a small number of proteins. The mitogenome encoded mRNAs are transcribed and translated by a distinctive mitochondrial protein-synthesizing system, some of whose components are specified by the mitochondrial genome. Characteristically, mitogenomes encode for rRNAs, while tRNAs and ribosomal proteins can be encoded for in the nucleus (Lang et al., 1999). The mitogenomes of Apicomplexa are characterized by their extreme coding reductionism, harboring only highly fragmented rRNAs, and *cox1*, *cox3*, and *cob* (Mathur et al., 2021). Protein coding genes are also highly fragmented in cyst-forming coccidians. In this section we succinctly (and certainly not exhaustively) describe a number of known molecular mechanisms underlying transcription and translation of apicomplexan mitogenomes genes.

We note that the complexity and breadth of nuclearly encoded proteins that significantly contribute to mitochondrial function -including protein import, organelle fission, other metabolic pathways, etc.- is by no means sufficiently reviewed in this section. Interested readership in the subject can find excellent recent reviews on the subject which are beyond the scope of the present work. Here, we highlight feature and mechanisms occurring in apicomplexans and other alveolates to showcase the diversity of processes that have been selected through thier evolution (Figure 1), and propose plausible mechanisms underlying the apicomplexan peculiarities.

### Mitochondrial Electron Transport Chain Complexes and Mitoribosomes

Recent “complexome” profiling has shown that the respiratory chain complex IV in *T. gondii*, which in humans and yeast houses *cox1* and *cox3*, contains peptides of these proteins, and 17 other divergent Apicomplexa-specific proteins (Maclean et al., 2021). Likewise, complexome profiling in *Plasmodium* revealed both divergent and clade-specific additions to all respiratory chain complexes, and expression of Cox1 and Cox3 peptides (Evers et al., 2021).

While the majority of mitochondrion-resident proteins are encoded for in the nucleus and imported, genes encoded for in the mitogenome are necessarily translated by mitochondrial ribosomes. Mitochondrial translation has been shown essential for survival in *P. falciparum* (Ke et al., 2018). Cross-species comparisons amongst Apicomplexa, alga and diatoms revealed that organellar ribosomes (i.e. of the mitochondrion and the apicoplast) have species-specific composition. In addition, they have undergone reduction, losing several small and large subunit proteins, and divergence both in sequence and ortholog length, with respect to bacterial ribosomes (Ke et al., 2018). In fact, several subunits of conserved mitochondrial ribosomes, which are present in diatoms, are missing in Apicomplexa. It has been proposed that these reductions in the mitochondria could be partially rescued by dually targeted proteins of plastid origin to both the mitochondrion and the apicoplast.

Noteworthy, the mitoribosomes of *T. gondii* and *P. falciparum* were recently identified and characterized by genetic ablation of genes encoding for putative protein components (Lacombe et al., 2019). In *T. gondii* it was shown that three distinct proteins, encoded by the nucleus, belong to two distinct macromolecular complexes, possibly corresponding to the small and large subunits of the ribosome. Meanwhile, in *Plasmodium*, immuno-EM experiments showed that three putative protein components of mitoribosomes are closely associated with the cristae. Knockdown of these proteins hypersensitized parasites to inhibitors targeting Complex III and blocked the pyrimidine biosynthesis pathway (Ling et al., 2020).

### tRNAs

In addition to the presence of a mitochondrial ribosome, translation of mito-encoded proteins implies the need for an organelle resident protein translation system, which includes tRNAs. Characteristically, apicomplexan mitogenomes do not encode for tRNAs. In fact, this is a character common to all alveolates, which we discuss in further detail below. Northern blot experiments probing for nuclear-encoded tRNAs on purified mitochondrial fractions, showed that in *T. gondii*, tRNAs are indeed imported. All but the cytosol-specific initiator tRNAMet were shown to be present both in the cytosol and in the mitochondria. Absence of formylated tRNAMet suggests that this amino acid modification is not required for translation initiation in the *T. gondii* mitochondrion (Esseiva et al., 2004; Pino et al., 2010). In addition to importing tRNAs, *P. falciparum* has been shown to be equipped with a mitochondrion-dedicated aminoacyl-tRNA synthetase (aaRS), known as mFRS, responsible for charging tRNAs imported into the mitochondria with phenylalanine on site. Intriguingly, mFRS is exclusive to the malaria parasites within the phylum (Sharma and Sharma, 2015). In contrast, no mitochondrial aaRS has been identified in *T. gondii*, suggesting that all tRNAs are imported in their charged form (Pino et al., 2010).

### rRNAs

rRNA fragmentation is widespread in nature, and by no means an exclusive feature of the Apicomplexa mitochondrial genomes. In Alveolata, extensive rRNA fragmentation might have appeared before divergence of colponemids and myzozoans (see below for a more detailed discussion of rRNA fragmentation in alveolates). However, the fragmentation observed in *Plasmodium* is the most extreme reported, and we therefore highlight it as an illustrative -extreme- example of rRNA fragmentation in Apicomplexa.

In *Plasmodium* out of a total of 34 identified small RNAs, 20 were initially assigned to both rRNA genes (Feagin et al., 1997); seven more were described later. These fragments range from 23 to 200 nucleotides in size whereby 12 correspond to SSU RNAs (totaling 804 nt) and 15 to LSU RNAs (totaling 1,233 nt) (Feagin et al., 2012). Fragments are dispersed throughout the mitogenome, and are coded for in either strand. It is unclear how these small RNA fragments come together to form a ribosome (Hillebrand et al., 2018). However, it has been proposed, based on 3D structure simulations, that fragments could cluster, by secondary-pairing interactions, on the interfaces of the two ribosomal subunits as separate entities to form a functional ribosome (Feagin et al., 2012). In addition, clustered organellar short RNA fragments (cosRNAs) complementary to the 5′ ends of rRNA fragments have been identified. These cosRNAs could be a recognizable footprint by RNA-binding proteins (RBPs) which might aid in the assembling of the rRNA fragments into a functional ribosome (Hillebrand et al., 2018).

The size, nucleotide sequence and arrangements are almost completely conserved among *Plasmodium* and *Leucocytozoon*. Despite complete apicomplexan mitogenomes, not all sequences presumed necessary for functional rRNAs have been identified (Feagin et al., 1997). Such absences might be accounted for by functional relocation to the nucleus, and targeting of small rRNA fragments from the cytoplasm. As a precedent, mitochondrial import of cytosolic 5S rRNA has been demonstrated in mammals (Entelis et al., 2001).

rRNA fragmentation has also been well described in dinoflagellates (Jackson et al., 2007; Kamikawa et al., 2007; Nash et al., 2007; Slamovits et al., 2007). Common with apicomplexans, these small rRNAs are oligo-adenylated, and presumably assemble into complete ribosomes carrying these short extensions (Feagin et al., 1997; Jackson et al., 2007).

### Cox1, Cox3, and Cob

The precise mechanisms of translation of resident mitochondrial protein are still to be teased out in Apicomplexa. As mentioned above, this is likely carried out by a ribosome resident to the organelle, aided by fragments of rRNA possibly brought together by specialized nuclearly encoded RBPs, and a combination of charged imported cytosolic and *in situ* charged tRNAs. Transcription of the mitogenome is mediated by a resident mitochondrial RNA polymerase, which most likely is also involved in mitogenome maintenance (Suplick et al., 1990; Ke et al., 2012). Transcripts are generated as polycistronic units, followed by cleavage and individual transcript polyadenylation (Rehkopf et al., 2000) to yield the shorter RNAs which correspond to the protein-coding and rRNA genes (Ji et al., 1996). In *Plasmodium*, for example, genome length transcripts have been detected from both DNA strands (Suplick et al., 1990).

**FIGURE 2:**
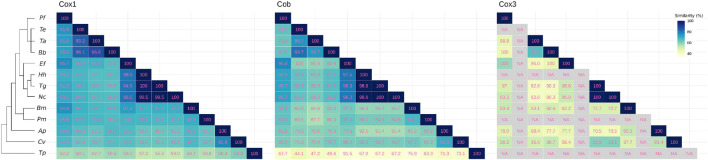
Protein sequence conservation of the three retained mitochondrial genes within Apicomplexa and evolutionarily related organisms. Heatmaps show similarity in local blastp pairwise protein alignments. Percentages of positive-scoring matches for Cox1, Cob, and Cox3, are shown according to the color scale, as indicated. Percentages of alignment coverage (alignment length normalized by the average protein length) are indicated as numbers. Abbreviated labels and NCBI accession identifiers for protein sequences used for Cox1, Cob, and Cox3, are as follows: Pf: *P. falciparum;* YP_009480320.1, YP_009480321.1, AAC06268.1; Te: *T. equi*, XP_025033546.1, XP_025033545.1, NA; Ta: *T. annulata*, XP_039113697.1, XP_039113699.1, XP_039113698.1; Bb: *B. bovis*, YP_001504106.1, YP_001504108.1, YP_001504107.1; Ef: *E. fuonis*, NC_039745.1, NC_039745, YP_009531492.1; Hh: *H. heydorni*, AFQ31671.1, AFQ31670.1, NA; Tg: *T. gondii*, AFQ31675.1, XP_018634734.1, AFQ31675.1; Nc: *N. caninum*, AFQ31673.1, QLF97291.1, QLF97290.1; Bm: *B. minutum*, BAS21362.1, BAS21364.1, BAS21363.1; Pm: *P. marinus*, ADZ98701.1, ADZ98702.1, NA; Ap: *A. peruviana*, AHA41651.1, AHA41650.1, AHA41680.1; Cv: *C. vietnamica*, AHA41634.1, AHA41615.1, AHA41635.1; Tp: *T. pyriformis*, AAA32102.2, AAD41943.1, NA. NA corresponds to missing or unavailable data. To the left, tree topology corresponds to that used for Figure 1.

*Cox*1 is the best conserved of all three protein coding genes amongst Apicomplexa (Feagin, 1994). The *Plasmodium* gene codes for the shortest *cox1* known, lacking the last 45 amino acids of the mammalian *cox1*. *Cob* is also highly conserved amongst species (Feagin, 1994) and, together with *cox1*, they have been used as molecular markers for phylogenetic analysis at different taxonomic levels. On the contrary, *cox3* is not well conserved (Feagin, 1994) and it is not present in all alveolates -it has been lost in ciliates (Burger et al., 2000). Nucleotide sequence identity has been shown for Hematozoa (He et al., 2014). Protein identity values for these three genes in different taxa representative of Alveolata are shown in Figure 2. It consistently shows that *cox1* is the most conserved gene, followed by *cob*. On the other hand, *cox3* shows overall low identity values. In addition, its coding region has not yet been identified for some taxa (in which they are expectedly present). For these proteins the minimum percentage of identity within Apicomplexa *sensu stricto* is 65, 59, and 42 (for Cox1, Cob, and Cox3, respectively), and these minimum percentages fall to 53, 35, and 42 within Alveolata. Of interest, Figure 2 also depicts the extension of the pairwise local alignments. It can be seen that, for Cob and Cox1, the conservation extends over almost the entire protein length and it is not limited to specific domains. Contrarily, for *cox3*, conservation is markedly reduced and in some cases, homology can be established only for specific fragments or domains (less than 20% of the length).

Initiation codons are still uncertain in *cox1* and *cox3*. Only the *cob* in *Leucocytozoon*, *H.* (*Haemoproteus*), and *Plasmodium* has an ATG triplet at the 5′ terminus that could be its initiation codon. The presence of alternative initiation codons has been determined for the *cob* genes in *H*. (*Parahaemoproteus*) and *Hepatocystis*. In these, ATT (Ile) or ACT (Thr) and AGT (Ser) substitute the canonical ATG, respectively (Pacheco et al., 2018). Similarly, the presence of canonical stop codons is seemingly reduced in apicomplexan and alveolate mitogenomes, with frequent loss of the TAA stop codon. A common occurrence is the rescue system using oligo-adenylation following a U nucleotide which creates an in-frame standard stop codon (TAA). This has been shown to happen in the *cox3* transcripts of all dinoflagellates analyzed (Waller and Jackson, 2009).

The redefinition of ORF boundaries, with apparent abandonment of standard start and stop codons is presumably observed in all alveolates (Edqvist et al., 2000; Waller and Jackson, 2009). In absence of RNA editing mechanisms, novel mechanisms are expected to either allow alternative start codons or the recognition of tRNA Met of non-standard initiation codons as Met codons. In either case, an unusual degree of flexibility during the initiation phase of translation is expected. It is interesting that, at least in ciliates, where tRNA Met is encoded in the mitogenome, it shows a truncated D stem and loop and unusual structural features in the anticodon loop, which might allow a more flexible codon-anticodon pairing to accommodate initiation codons that are variants of AUG (discussed in Edqvist et al., 2000). Conspicuously, as mentioned above, cytosol-specific initiator tRNAMet is undetectable in the *T. gondii* mitochondria.

When stop codons are absent or cannot be formed by oligo-adenylation, active mechanisms are needed to avoid translation arrest when the ribosome arrives at the undefined transcript termini. One such mechanism in eukaryotes involves the protein Ski7p and an equivalent system in prokaryotes employs a transfer messenger RNA. It is conceivable that alveolates have adopted components of either of these two rescue systems for regular translation termination in the mitochondrion, in order to avoid transcripts and proteins being targeted for degradation (Waller and Jackson, 2009).

Out of all apicomplexan mitogenomes, the cyst forming coccidians pose the most challenging scenario to understanding transcriptional regulation. RT-PCR experiments in *T. gondii* by Namasivayam et al. (2021) detect nearly full-length *cob* and *cox3*, and a partial *cox1.* Whether these are exclusively generated from the low abundance full length coding sequences detected by ONT (Namasivayam et al., 2021), or are alternatively or additionally generated by an underlying mechanism of recombination of the highly fragmented ORFs coding for Cox1, Cox3, and Cob, remains to be deciphered. An interesting finding of Namasivayam et al. (2021), is that many ORFs were shown to be followed by variant polyadenylation signals which may affect the detection of full length transcripts by Illumina based RNA-seq as sequencing libraries are typically generated following oligo(dT) purification (Namasivayam et al., 2021).

Long reads have been widely used to resolve or enhance nuclear genomes of various parasites. Since they are orders of magnitude smaller and simpler, complete mitochondrial genomes have surged from these same sequencing projects. However, there are notable exceptions; the mitochondrial genome of coccidians being a beautifully illustrative example. From PacBio and ONT sequencing the number of nuclear chromosomes of both *T. gondii* and *N. caninum* was reduced from 14 to 13, and important chromosomal rearrangements between both species were described (Berná et al., 2021; Xia et al., 2021). However, none of the mitochondrial genomes could be completely resolved; neither have been conclusively resolved the level at which the diversity exists nor the mechanisms underlying this diversity. For these complex mitogenomes, as can also be the case for *Sarcocystis*, additional strategies must be adopted. In particular, better coverage and more robust results are necessary. Direct RNA long reads emerge as a promising alternative to shed light onto the undescribed diversity and perhaps new transcriptional mechanisms in these parasites.

With this in mind, we explored the recently published direct ONT mRNA data from *T. gondii* (Lee et al., 2021). We searched for clues of the underlying transcription mechanism of gene fragments in the *T. gondii* mitogenome. Although this sequencing was performed to identify possible isoforms in the nuclear genes (Lee et al., 2021), a total of 355 ONT reads (from 112 to 2,962 bp long) mapped in the mitogenome of *T. gondii*. The longest mRNAs correspond almost exactly to the series of discrete sequence blocks described by Namasivayam et al. (2021). We note that this dataset had been analyzed by Namasivayam et al. (2021) prior to publication. While the authors reached an akin conclusion, a markedly lower number of reads mapped to the individual sequence blocks and their combinations. Thus, the significantly higher number of reads mapped here further support the mechanism suggested by Namasivayam; ordered blocks are indeed transcribed in the required order so as to produce a full length mRNA (Namasivayam et al., 2021).

**FIGURE 3:**
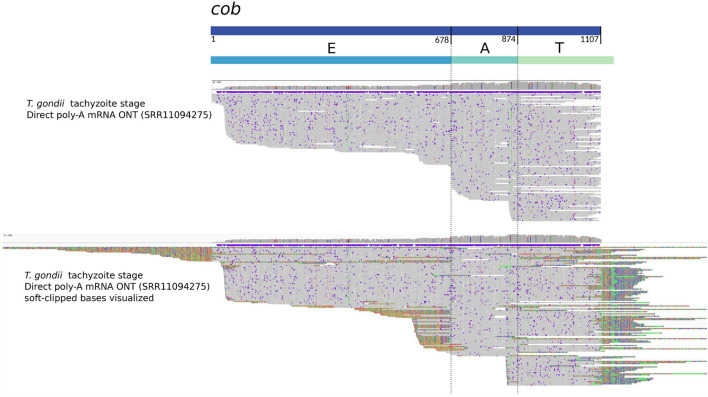
Mapping of Oxford Nanopore mRNA reads with respect to the *cob* sequence in the mitogenome of *T. gondii*. The figure shows an adapted visualization obtained with IGV (Thorvaldsdóttir et al., 2013) of ONT direct RNA reads (Lee et al., 2021) aligned with minimap2 (Li, 2018) to the *cob* sequence inferred for *T. gondii*. Above, the rectangles represent the *cob* gene sequence and the proposed constituent sequence blocks “EAT” (Namasivayam et al., 2021), respectively. Vertical lines highlight the limits between E and A, and A and T blocks. In the alignment section, the upper part shows ONT reads aligned with hard clipping, and the bottom shows the same ONT reads with soft clipping visualization. Notoriously, there are reads covering the complete *cob* gene, but others align only to one (T) or two blocks (AT). In the case of the visualization with soft-clipping, the aligning part of the reads extend to sequence contexts not contiguous but otherwise present in other parts of the mitogenome. The latter correspond to other sequence blocks.

However, the three protein-coding genes encoded for the *T. gondii* mitogenome are formed by a minimum of three sequence blocks. Interestingly, we and Namasivayam and colleagues, found reads supporting the existence of transcripts corresponding to single blocks, or the combination of only two blocks for *cob*. Several of these mRNA reads, as shown in Figure 3, do not extend beyond, nor do they include other sequence blocks. Instead, they are truncated in the exact same position, coinciding with the block’s boundary. To us, this suggests that an unidentified post-transcriptional mechanism may exist to precisely cleave the individual block sequences from their primary polycistronic mRNA. One could also envision the possibility that these expressed fragments are somehow additionally combined *a posteriori* to form a complete messenger, thus originating a mature and complete mRNA ready for translation. In fact, we propose that the existence of such mechanisms could even be needed to compensate for the low abundance of full-length transcripts originating from a only handful of sequence block combinations coding for the complete ORFs in the mitogenome.

### Post-transcriptional Processing in Alveolata

Mitochondrial genomes in *Plasmodium* and piroplasmids seem to produce mRNA transcripts that are more or less ready for translation. In *Toxoplasma* and *Neospora*, one complete gene copy could produce all full length mRNAs and/or transcript fragments might be further processed to obtain full length messenger RNAs. While this is unknown, some clues can be obtained from what has been described in other alveolate lineages. Thus, we summarize some post-transcriptional oddities to highlight the diversity of molecular processes that could be at hand for these protists.

First, apart from the rRNAs fragments, additional genes have been found split. In *Paramecium* and *Tetrahymena*, the *nad1* gene is split in two portions that are encoded for and transcribed from different DNA strands. Edqvist et al. (2000) determined that the corresponding transcripts are not trans-spliced, thus separate N- and C-terminal portions of Nad1 are independently produced in these systems. Whether the separate polypeptides can associate to generate a bipartite, functional Nad1 protein -analogous to what occurs with the rRNA fragments- or some other type of covalent joining occurs at the protein level, remains unclear. In dinoflagellates, *cox3* is split in two fragments, each producing a polyadenylated transcript. To achieve a mature mRNA, the fragments are joined when a certain number (taxon specific), of A nucleotides of the oligo(A) tail from the 5′ major transcript are incorporated (Jackson et al., 2007), implying a novel mechanism for splicing in dinoflagellate mitochondria. The inserted adenosine nucleotides in turn result in one or more lysines in the Cox3 protein, representing an hydrophilic insertion which is tolerated due to its position between the transmembrane domains (Jackson and Waller, 2013). *Trans*-splicing is present in the mitochondrial genome of plants. Autocatalytic molecular events are mediated by sequence flanking the precursor elements, which resemble group II introns (Bonen, 1993; Edqvist et al., 2000). To our knowledge, no such elements have been identified flanking genes in alveolates. On the other hand, in mycorrhizal fungi, there is evidence that the RNA from a fragmented *cox1* gene is joined by flanking exon sequences that form a group I intron structure, potentially in conjunction with the *nad5* intron sequences. The latter acts as an additional helper (Nadimi et al., 2012). Additional third helpers are also used in *Chlamydomonas* (Goldschmidt-Clermont et al., 1991). Though purely speculative, the possibility of small RNAs produced in *trans* being involved in the resolution of trans-splicing events is appealing to propose as a putative mechanism operating in Apicomplexa. These could be coded for by the nucleus and imported into the mitochondrion to aid in post-transcriptional processing of mitochondrial transcripts.

In addition to split of mitogenome genes, multiple mitochondrial gene fusions have been reported in early branching dinoflagellates (*cob-cox3*) and chromerids (*Chromera*: *cox3-cox1*; *Vitrella*: *cob-cox1*) (Slamovits et al., 2007; Oborník and Lukeš, 2015). As proteins are part of different electron transport complexes, they must function separately. It is unknown if the processing mechanism -shared or not in both lineages- involves cleavage of the initial transcript, independent translation from the bi-cistronic transcript or post-translational cleavage.

Third, the most extensive case of translational frameshifting was determined for *cox1* mRNA in *Perkinsus*, a sister taxon to Dinoflagellata. From the DNA sequence it can be inferred that the reading frame must be shifted ten times, at every AGG and CCC codon, to yield a consensus Cox1 protein (Masuda et al., 2010). Whether the mechanism involves ribosomal frameshifts and/or specialized tRNAs, is unknown. No evidence of an akin mechanism operating in Apicomplexa has been reported.

Finally, RNA editing can explain differences between nucleotide sequences at the DNA and mRNA levels. All mitochondrial protein-coding genes and some rRNA fragments are edited in dinoflagellates, where nine of twelve possible substitutions have been observed and concern up to 6% of the nucleotides in transcripts (Lin et al., 2002; Waller and Jackson, 2009). The mechanisms underlying this unprecedentedly versatile editing are unknown. However, with the sole exception of core dinoflagellates, RNA editing is absent from most alveolates making it unlikely for this to be the adopted mechanism used by cyst forming coccidians to make sense of their fragmented genomes.

## How Peculiar Are Mitogenomes in Apicomplexa?

The progression of a free-living alpha-proteobacterium into a symbiotic mitochondrion necessarily involves a history of decayment. To fully grasp how peculiarly reductive the apicomplexan mitogenomes are we analyze the general features of mitochondrial genomes in additional Alveolata lineages, providing an expanded comparative evolutionary framework.

Apart from apicomplexans, alveolates comprise the well-studied dinoflagellates and ciliates - and *Perkinsus*. These major lineages have evolved distinct and peculiar characteristics. However, the reconstruction of ancient transitions cannot be fully understood unless deep-branching lineages, which retain ancestral characteristics, are incorporated into the discussion. As such, it becomes relevant to highlight inferences drawn from the recently described *Colponema* and *Acavomonas* alveolate lineages. Lastly, we revisit the mitogenome features of chromerids, a sister lineage to Apicomplexa, consisting of coral-associated photosynthetic protists.

To date, about a dozen mitogenomes have been described for ciliates (Burger et al., 2000; Edqvist et al., 2000; Park et al., 2019). They are linear and relatively large (up to 80 kb), with repeat elements at the ends of the linear DNA (telomeres). They present a variable number of protein coding genes involved in aerobic respiration, an unusually high number of unassigned ORFs -many of them being ciliate-specific, two bipartite rRNAs (LSU and SSU) and a few tRNAs. According to this, they contain more than the standard set of genes encoded by mitochondrial genomes in other organisms but less tRNAs. Interestingly, as mentioned before, ciliates do not encode for *cox3* but harbor additional NAD dehydrogenase subunit genes (*nad7*, *nad9*, and *nad10*). No intron sequences have been reported. In addition to the standard ATG, start codons include: ATA, ATT, GTG, and TTG. The latter makes it challenging to assign initiation codons, raising questions about the underlying mechanisms of gene expression. Only TAA is used to terminate translation, while TGA, as in many other mitochondrial translation systems, specifies tryptophan. To note, ciliate mitogenomes harbor, in addition to the rRNA genes, a *nad1* gene which is split and rearranged, whereby N-terminal and C-terminal portions are transcribed from different strands, and translated separately (Burger et al., 2000; Edqvist et al., 2000).

Curiously, dinoflagellates exhibit an evolutionary trend toward mitogenome simplicity, all the while increasing their complexity (Gagat et al., 2017). As in apicomplexans, mitogenomes of several dinoflagellates code for *cox1*, *cox3*, and *cob*, and display the two fragmented rRNAs. No tRNAs have been identified implying, as in Apicomplexa, complete reliance on imported tRNAs for protein translation. None of the NADH dehydrogenase genes (*nad1*, *nad4*, and *nad5*) have been observed in dinoflagellate suggesting that the loss of complex I has occurred before lineage divergence (Waller and Jackson, 2009; Flegontov and Lukeš, 2012). However, mitogenomes consist of multiple linear chromosomes with sizes ranging 6–10 kb, and even longer. The mitogenome of *Symbiodinium minutum*, a reef-building coral endosymbiont, for example, totals 326 kb; 99% of which is composed of non-coding sequences (Shoguchi et al., 2015). The large size and complexity of the dinoflagellate mitogenome is owed to numerous amplification and recombination events; not only does this impact genome structure, but also its gene content. Similarly to what is observed in coccidians, dinoflagellates display multiple copies of each gene, and gene fragments linked in numerous configurations (Nash et al., 2008). Some of the dinoflagellates gene features are shared with other alveolates; for instance, the usage of unconventional start codons or use of polyadenylation to generate stop codons is an alveolate-wide observation.

Other features, however, are peculiar to the lineage. Specifically, dinoflagellates exhibit RNA editing and *cox3* processing. RNA editing occurs in the transcripts of all protein-coding genes and in some rRNA fragments. Editing is absent in *Oxyrrhis marina*, an early branching dinoflagellate. However, the younger the genus, the greater the versatility and scale of changes that occur through RNA editing (Waller and Jackson, 2009). The *cox3* gene is broken between regions coding for the sixth and seventh transmembrane helices, in all core dinoflagellates. In order to create the mature mRNA, a certain number of adenosine nucleotides are added between both primary transcripts. This in turn results in one or more lysines in the Cox3 protein (Jackson and Waller, 2013). Finally, another peculiarity of early *O. marina*, is the *cob-cox3* gene fusion (Slamovits et al., 2007). The mechanisms to manage this gene fusion and its products are currently unknown.

*Perkinsus* comprises species that affect many bivalve mollusk host species globally. Current evidence supports that, together with other taxa, they constitute the separate phylum Perkinsozoa, sister to Dinoflagellata (Mangot et al., 2011; Janouškovec et al., 2017). Mitogenomes have been assembled for some species; their size, topology and gene content resemble those described in dinoflagellates (Bogema et al., 2021). However, a few differences are attention worthy. First, the *cox3* gene is undetectable. In this sense, the phylum is more similar to ciliates. Secondly, there exists a peculiar *cox1* gene; the reading frame must be shifted ten times, at every conserved AGG and ACC codon, to yield the consensus Cox1 protein (Masuda et al., 2010; Bogema et al., 2021). The mechanisms needed to couple with this translational frameshifts are unknown but an active and efficient machinery is proposed to be required (Masuda et al., 2010).

*Colponema* and *Acavomonas* belong to two predatory alveolate phyla that lack secondarily derived morphological characteristics and retain cytoskeletal characteristics of the ancestral aveolate. Thus these phyla are pivotal to our understanding of alveolate evolution (Janouškovec et al., 2013; Tikhonenkov et al., 2014). Inferences strongly support *Acavomonas* as sister to myzozoans and *Colponema* branching deeper, near the base of alveolates or sister to ciliates (Janouškovec et al., 2013). In these organisms, mitochondria are oval and have tubular cristae. The *Acavomonas* mitogenome is a 50.4 kb single linear chromosome with terminal inverted repeats (TIRs), and 38 bp tandem telomeric repeats at the ends. This linear monomeric organization ending in telomeres resembles features described in ciliates, suggesting this represents the ancestral state in Alveolata. While this organization has changed in dinoflagellates and in most apicomplexans, piroplasmids may retain the ancestral state (Janouškovec et al., 2013).

The *Acavomonas* mitogenome has retained the largest gene set among all alveolates, including 14 genes previously unknown in the group. While ciliates are somewhat tRNA gene poor, *Acavomonas* has retained 21 mitochondrial-encoded tRNAs, and requires import of only three tRNA species. In this sense, it appears that independent loss of tRNA genes with concomitant import of aminoacylated tRNAs from the cytosol and use of alternative NADH dehydrogenase, has driven the main gene content reduction in myzozoans. The ancestral alveolate mitogenome was therefore tRNA rich, and reduction in ciliates and myzozoans occurred independently (Janouškovec et al., 2013). Interestingly, large and small rRNA genes are fragmented into multiple pieces, but the *nad1* gene is complete. The bipartite rRNAs and *nad1* are particularities of ciliates and fragmented genes appeared independently in myzozoa (instead, *nad5* is split into two fragments that are widely separated in the genome). *Acavomonas cox2* gene is intact and encoded for in the mitogenome, thus its split and migration to the nucleus is an event that occurred in the ancestor of myzozoans. Other gene peculiarities in ciliates, such as *cox3* absence, are not present in this lineage, suggesting that ciliates harbor many peculiarities that were not present in the alveolate ancestor.

Given their phylogenetic position within Alveolata, the deciphering of the chromerids *C. velia* and *V. brassicaformis* genomes have been instrumental in shedding light onto some conundrums about the evolution of obligate apicomplexan parasites from the free-living phototrophic algae (Woo et al., 2015). Their mitochondrial genomes have been reported (Flegontov et al., 2015) and, together with the analysis of mitochondrial pathways reconstructed from their nuclear genomes, they point to an unprecedented reduction of the respiratory chain in *C. velia*, an aerobic eukaryote. *Chromera* lacks respiratory complexes I and III, whereas *Vitrella* and apicomplexans are missing only complex I (Flegontov et al., 2015; Oborník and Lukeš, 2015). *Chromera* mitochondrial DNA is composed of short linear molecules and similarity searches identified a single conserved gene *cox1* and a highly divergent *cox3* gene. However, these genes appear multiple times in different mitogenome contexts. What is more, *cox3* seems to be fused with an upstream *cox1* fragment, and a fusion transcript is produced which apparently undergoes a subsequent cleavage. Unexpectedly, *cob* gene was absent from the assembly and original reads. Fragments of rRNA genes were also found. No RNA editing was determined. *Vitrella* mitogenome showed similar characteristics but, regarding its protein-coding gene content, it contains a fused *cob-cox1* gene and a divergent *cox3*.

The common features and oddities of the mitogenomes in Alveolata are summarized in Figure 1. Based on the mitochondrial genome features of ciliates (with exclusion of particular oddities) and *Acavomonas*, it can be hypothesized that the common ancestor of alveolates carried a mitogenome of about 50 kb, in a single linear chromosome, ending in TIRs and telomeres. The ancestor genome was protein-coding and -relatively- tRNA gene rich. Canonical start and stop codons were not always present. The two rRNA genes may have been complete, or not, but the direct ancestor of *Acavomonas* likely acquired fragmented rRNA genes. At the common ancestor of Myzozoa, a spectacular genome and gene content reduction occurred, arguably representing the largest and most profound reduction discovered in any aerobic mitochondrion thus far. Only three protein coding genes -*cox1*, *cox3*, and *cob* were retained; all tRNA genes were lost with the concomitant gain of mechanisms for cytosolic tRNAs import. NADH dehydrogenase genes were irrecoverably lost. With certain differences and exceptions (further reductions in *Chromera*, some gregarines and *Cryptosporidium*), gene content has been maintained more or less constant throughout myzozoans. In contrast, the evolutionary history of the group is marked by changes in the mitogenome size, organization and topology which seems to have evolved bimodally, with two opposite possible configurations. The first configuration is represented by the smallest known mitogenomes, those of haemosporidians and piroplasmids. They are small linear monomers, with telomeric ends and can be found forming linear concatemers or alternative conformations. This configuration seems to be more parsimonious as the default state in the common ancestor of Myzozoa thus it might be retained in apicomplexans such as *Plasmodium* and *Theileria*. The second configuration is present in dinoflagellates, *Chromera*, *Vitrella*, and also coccidians, and involves an expanded, fragmented and scrambled state of the mitogenome, possibly due to extensive recombination. While this configuration was already known for dinoflagellates and chromerids, it has only been recently described for *Toxoplasma* and *Neospora* (Berná et al., 2021; Namasivayam et al., 2021). In fact, for coccidians, additional details on the “21 building sequence blocks” are only beginning to emerge. While it is inferred that these seemingly messy mitogenomes have arisen recurrently (at least three times), it is unknown whether they share the same underlying molecular mechanisms of emergence. What is more, whether coccidian sequence blocks are mirrored in the other myzozoans is unknown. Finally, what the selective forces operating in these extraordinary complex mitogenome architectures are, remains a mystery.

In this sense, new long read technologies will be instrumental in shedding light onto the unknown aspects of structure and function of the mitogenomes in Apicomplexa, particularly illuminating our understanding of the possible transcriptional and post-transcriptional mechanisms underlying the survival of apicomplexans, in light of their unconventional mitogenomes and parasitic lifestyles.

## Author Contributions

LB, NR, and MF contributed to conception and design of the manuscript. All authors contributed to manuscript revision, read, and approved the submitted version.

## Conflict of Interest

The authors declare that the research was conducted in the absence of any commercial or financial relationships that could be construed as a potential conflict of interest.

## Publisher’s Note

All claims expressed in this article are solely those of the authors and do not necessarily represent those of their affiliated organizations, or those of the publisher, the editors and the reviewers. Any product that may be evaluated in this article, or claim that may be made by its manufacturer, is not guaranteed or endorsed by the publisher.
